# Lower Extremity Injuries in Adult Pickleball Players: A Systematic Review of Injury Types, Mechanisms, and Risk Factors

**DOI:** 10.7759/cureus.108841

**Published:** 2026-05-14

**Authors:** Ashley Okhovat, Eric Ferkel, Sherese A Richards

**Affiliations:** 1 Biomedical Education, California Health Sciences University-College of Osteopathic Medicine (COM), Clovis, USA; 2 Orthopedic Surgery, Southern California Orthopedic Institute, Los Angeles, USA

**Keywords:** achilles tendon, fractures, lower extremity injuries, older adults, pickleball, sports injuries, systematic review

## Abstract

Pickleball is one of the fastest-growing sports in the United States, particularly among adults aged 50 and older. As participation has increased, so have reports of musculoskeletal injuries, with the lower extremity frequently identified as the most commonly affected region. Despite growing clinical awareness, no systematic review has comprehensively examined the types, mechanisms, and risk factors of lower extremity injuries among adult pickleball players.

A systematic review was conducted following Preferred Reporting Items for Systematic Reviews and Meta-Analyses (PRISMA) guidelines. PubMed, MEDLINE, EMBASE, and Google Scholar were searched for peer-reviewed articles published between January 2004 and March 2025 using terms and Boolean combinations related to pickleball and lower extremity injuries. Studies were included if they reported pickleball-specific injury data in adults aged 18 years or older, addressed lower extremity injuries, and discussed injury types, mechanisms, or risk factors. Fifteen studies met the inclusion criteria and were independently reviewed by all authors.

Sprains, strains, and fractures were the most commonly reported injury types, with Achilles tendon rupture also notably represented. Falls were the leading mechanism of injury, followed by running or lunging forward and foot or ankle inversion. Older age was a consistent risk factor across studies. Age 50 years or older was a consistent risk factor across nearly all studies. Gender-based trends were mixed: males were more frequently injured overall, but females were disproportionately affected by fractures (p < 0.001).

Lower extremity injuries in pickleball are common, predominantly involve sprains and strains and fractures, and disproportionately affect older adults. Falls and rapid directional movements are the primary injury mechanisms. Clinicians, community organizations, and facility operators should prioritize implementation of structured injury prevention programs that include dynamic warm-up, proprioceptive training, sex-specific safety messaging, sport-specific footwear guidance, and routine court surface inspection.

## Introduction and background

Pickleball, a rapidly growing paddle sport in the United States, has seen a parallel rise in reported musculoskeletal injuries, particularly involving the lower extremities [1-6]. Designed as a low-impact, paddle-based game combining elements of tennis, badminton, and table tennis, pickleball is especially popular among individuals aged 50 and older [1,2]. While the sport promotes cardiovascular health, social interaction, and overall fitness, it also carries a notable risk for injury, often underestimated by recreational players [3-5].

Lower extremity injuries are among the most frequently reported musculoskeletal events in adult pickleball players. Ankle sprains and strains are the most common subtype, followed by fractures of the ankle, foot, and lower leg, and less frequently by Achilles tendon ruptures, which are notably represented among elderly participants [2,3,5-10]. Mechanistically, these injuries occur during dynamic movements such as sudden changes in direction, lunging, pivoting, and foot or ankle inversion (an inward rolling of the foot at the ankle that commonly produces lateral ligament strain or fracture). External factors, including slips, trips, and falls, are additional major contributors [2,6,10,11]. Many of these injuries occur in players without formal training in warm-up techniques, proper footwear selection, or injury prevention strategies [2,11].

Lower extremity injuries carry particular clinical importance in this demographic. They frequently require imaging or surgical intervention, are associated with prolonged recovery relative to upper extremity injuries, and can result in lasting loss of independent mobility in older adults; each of these outcomes carries significant functional, psychological, and economic consequences for an aging population [12,13].

Risk factors in this population include advancing age, prior musculoskeletal injury, inadequate conditioning, and insufficient pre-activity warm-up [1-11,14-15]. Gender-based trends have also emerged: males tend to sustain more soft tissue injuries, including muscle strains and Achilles tendon ruptures, while females are disproportionately affected by fractures, likely related to differences in bone density and biomechanics [3,8,14,15].

Treatment patterns vary by injury type. Non-operative management is typically employed for sprains, strains, and minor fractures [3,6]. In contrast, Achilles tendon ruptures are more frequently treated with surgical repair, with reported surgical rates ranging from 60% to 88.1% [6,10]. The rising popularity of pickleball has also led to a marked increase in healthcare utilization, including emergency department visits and hospitalizations [4,15].

Despite growing awareness, few studies have systematically examined the full spectrum of lower extremity injuries in adult pickleball players, particularly with attention to injury mechanisms, risk factors, and sex-based differences [11,16]. Given the expanding player base and associated public health impact, a deeper understanding of these trends is critical for guiding prevention, treatment, and policy efforts. To address these gaps in the literature, we systematically examined the types, mechanisms, and risk factors associated with lower extremity injuries in adult pickleball players over the past two decades.

## Review

Study design 

This systematic review was conducted in accordance with the Preferred Reporting Items for Systematic Reviews and Meta-Analyses (PRISMA) guidelines [17]. The review protocol was not prospectively registered.

Search strategy 

A comprehensive search of electronic databases, including PubMed, MEDLINE, EMBASE, and Google Scholar, was conducted for peer-reviewed articles published between January 2004 and March 2025. Search terms and representative Boolean combinations included: ("pickleball" AND ("lower extremity" OR "foot" OR "ankle" OR "knee" OR "Achilles" OR "fracture" OR "sprain" OR "strain")); ("pickleball" AND ("musculoskeletal injury" OR "injury mechanism" OR "injury epidemiology")); and narrower terms such as "pickleball Achilles rupture," "pickleball foot injury," and "pickleball musculoskeletal injury patterns." Filters were applied for English language, human studies, and publication date within the specified range.

Study selection 

After removal of duplicates and non-relevant titles, articles were screened based on title and abstract. Full texts were then reviewed to determine eligibility based on predefined inclusion and exclusion criteria. Initially, the search targeted studies directly aligned with lower extremity pickleball injuries. Due to the limited number of publications specifically addressing this anatomical focus, the scope was broadened in two deliberate ways: (1) studies addressing general pickleball injuries were included when extractable lower extremity data were present within the broader analysis, and (2) one narrative review was retained because it provided mechanistic data on sport-specific injury patterns not reported elsewhere. All articles were independently reviewed by all authors, with screening decisions made through consensus. Holistic judgment was applied in cases where studies partially met inclusion criteria or contained extractable data relevant to lower extremity injuries within a broader analysis.

Inclusion criteria 

Studies were included if they met the following criteria: (1) participants were adults aged 18 years or older; (2) the study reported pickleball-specific injury data; (3) the study addressed lower extremity injuries or contained extractable lower extremity data within a broader injury analysis; (4) the study was a primary research study, systematic review, scoping review, or narrative review containing original data synthesis or extractable mechanistic data; and (5) the study discussed injury types, mechanisms, or risk factors. 

Exclusion criteria 

Studies were excluded if they focused exclusively on pediatric populations, reported only on general racquet sports without pickleball-specific data, addressed only upper-extremity or head injuries without lower-extremity data, were opinion pieces or editorials without original data, or focused solely on treatment or prevention without reporting injury epidemiology.

Data extraction and synthesis 

Data extracted from each included study encompassed study design, sample size, population characteristics, injury types, anatomical regions affected, mechanisms of injury, risk factors, and reported statistical findings. While a formal risk of bias assessment instrument was not applied, each study was qualitatively evaluated by all three authors for methodological transparency, clarity of outcome definitions, and relevance to the review question prior to inclusion. Discrepancies were resolved through discussion and consensus. Statistical findings reported in the included studies (for example, p-values and proportions) were extracted and summarized descriptively; no pooled estimates or meta-regressions were performed. Due to heterogeneity in study designs, outcome measures, and reporting methods, a meta-analysis was not feasible. Findings were synthesized narratively and organized by thematic categories: risk factors, injury types, affected anatomical regions, and mechanisms of injury.

Results 

Study Characteristics 

**Figure 1:**
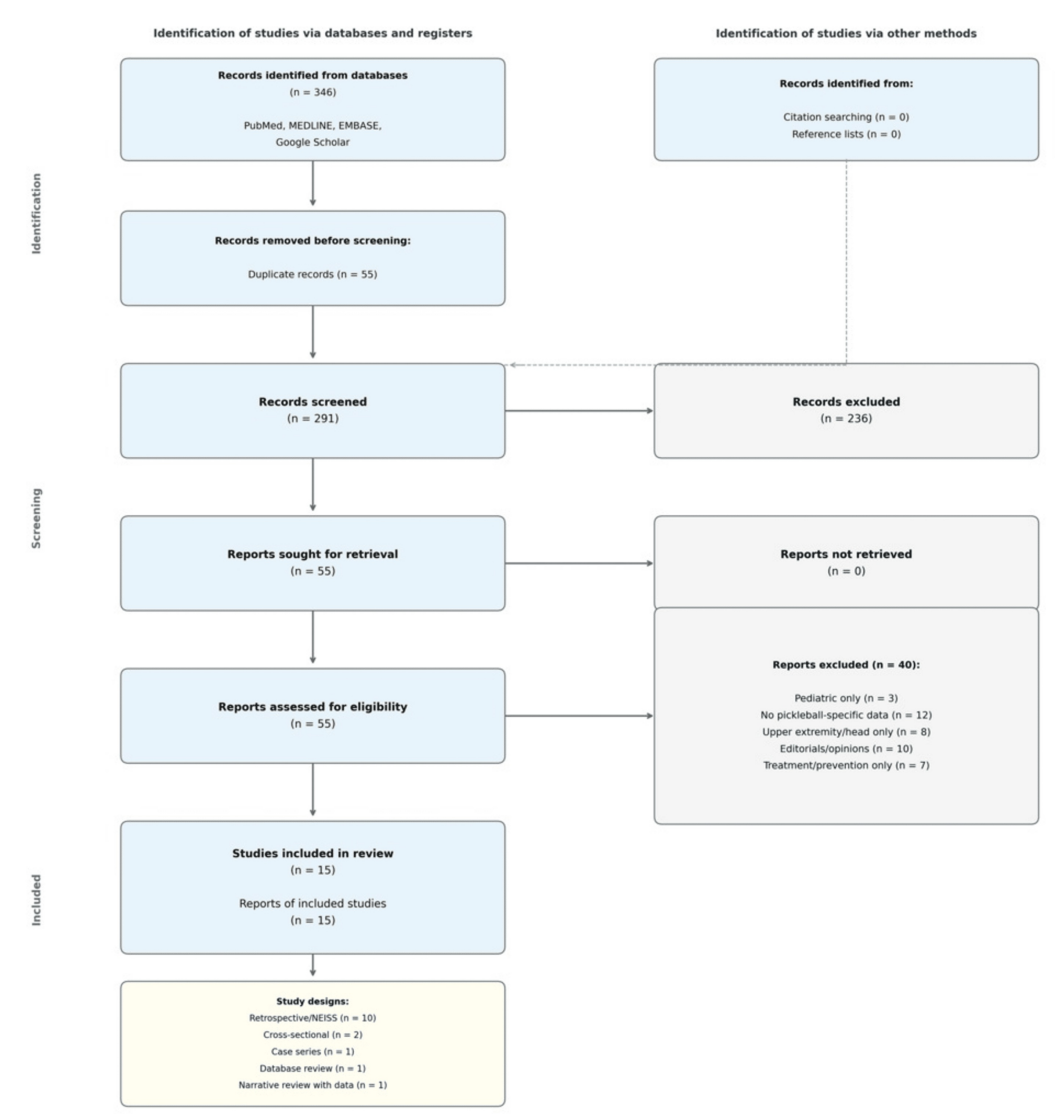
PRISMA flow diagram illustrating the study selection process. A total of 346 records were identified, 291 remained after duplicate removal, 55 underwent full-text review, and 15 met the inclusion criteria for qualitative synthesis.

The systematic search identified 346 records across all databases. After removal of duplicates and screening of titles and abstracts, 55 articles were assessed for eligibility. Following full-text review against inclusion and exclusion criteria, 15 studies were included in the final analysis (Figure 1, Table 1).

**Table 1:** Characteristics of 15 included studies. ED: emergency department; EHR: electronic health record; EMR: electronic medical record; ICD: International Classification of Diseases; LE: lower extremity; NEISS: National Electronic Injury Surveillance System; UE: upper extremity.

Ref No.	Author (year)	Study design	Sample size	Data source	Primary focus	Key lower extremity findings	Limitations
[1]	Weiss et al. (2021)	Retrospective (NEISS)	n = 87,820	NEISS database (US EDs)	Senior pickleball vs. tennis injuries, 2010–2019	Sprains and strains and falls common; slip, trip, fall, and dive mechanisms predominant	Includes tennis comparator; restricted to seniors aged 60 and older; US EDs only
[2]	Kim et al. (2025)	Cross-sectional (survey)	n = 237	Self-reported survey	Injury epidemiology and risk factors in older players	Wrist 12.7%; females 69.1% injured (p < 0.001); falls 65.5%; muscle and tendon involvement 61.8%	Self-reported data; older population only
[3]	Herzberg et al. (2025)	Case series (EHR)	n = 487	Single-institution EHR	Pickleball injuries at a single center, 2017–2022	Rotator cuff 11%; calf 6%; back 7.8%; sprains and strains common; contusions noted	Single institution; broad injury scope rather than LE-specific
[4]	Cheng et al. (2025)	Retrospective (NEISS)	n = 15,891	NEISS database (US EDs)	Injuries and hospitalizations, 2020–2022	Substantial increase in injuries and hospitalizations; fractures approximately 29%	Broad injury scope; US EDs only
[5]	Yu et al. (2025)	Descriptive epidemiology (NEISS)	approx. 1,100 cases	NEISS database (US EDs)	Mechanisms and trends over 10 years	Sprains and strains 21%; fractures 29%; falls and inversion mechanisms; females fractures 32.7%	Broad injury scope; US EDs only; NEISS coding limitations
[6]	Opara et al. (2024)	Retrospective (EMR)	n = 166	Single orthopedic center	LE injuries: description, treatment, and return to play	Majority older than 60 years, male; treatment and return-to-play patterns characterized for LE injuries	Single center; greater than 50% lost to follow-up; includes paddleball
[7]	Forrester (2020)	Retrospective (NEISS)	n = 19,012	NEISS database (US EDs)	ED-treated pickleball injuries	Sprains and strains 28.7%; fractures 27.7%; LE 32.0%	Broad injury scope; ED narratives only
[8]	Ghattas et al. (2024)	Descriptive epidemiology (NEISS)	n = 397	NEISS database (US EDs)	Pickleball-related fractures, 2002–2022	Falls leading cause of fractures (p < 0.001); females sustained more fractures (p < 0.0001)	Fractures only; US EDs only
[9]	Jones and Hammig (2024)	Retrospective (NEISS)	n = 296	NEISS database (US EDs)	Age as a risk factor	LE 57.9%; age a significant risk factor (p = 0.019); fractures 30%	Broad injury scope; US EDs only; small sample
[10]	McCahon et al. (2024)	Retrospective case series	n = 2,684 total; n = 43 pickleball	Multi-site orthopedic registry	Achilles tendon injuries in elderly players	Pickleball patients significantly older; Achilles 39.4%; high surgical rate (88.1%)	Retrospective; ICD-coded; limited pickleball-specific sample
[11]	Gregg et al. (2025)	Narrative review	Not applicable	Literature synthesis	Mechanisms: slip, trip, fall, dive	Mechanisms characterized including falls, dives, and slips; sport-specific biomechanics discussed	No new data; narrative synthesis only
[14]	Boroumand et al. (2025)	Descriptive epidemiology (NEISS)	n = 749	NEISS database (US EDs)	Two-decade upper and lower extremity injury trends	LE injuries increased dramatically; adults higher LE frequency (p = 0.022); males more LE (p < 0.0001)	Broad injury scope; US EDs only
[15]	Kingston et al. (2024)	Retrospective (institutional)	n = 198	Institutional database	Foot and ankle injuries	Lunging 30.9%; planting 16.5%; inversion 15.5%	Foot and ankle only; single institution; ED narratives
[16]	Sellwood et al. (2025)	Retrospective database review	n = 43	Illuminate Insight imaging platform	Imaging patterns in pickleball injuries	LE 58%; UE 37%; trunk 5%	Small sample; single platform; limited demographics
[18]	Changstrom et al. (2022)	Descriptive epidemiology (NEISS)	n = 7,723	NEISS database (US EDs)	Racquet and paddle sports injuries, 2007–2016	UE injuries predominant (p < 0.001); radius fractures 30%	Includes multiple racquet sports; broad scope

Of the 15 included studies, 10 were retrospective studies using electronic medical records or the National Electronic Injury Surveillance System (NEISS) database [1-5,8-10,14,15], two were cross-sectional studies [1,2], one was a case series [3], one was a retrospective database review of imaging data [16], and one was a narrative review with extractable mechanistic data [11]. Study sample sizes ranged from 43 to 87,820 participants. The majority of studies (n = 11) were conducted in the United States, with data sources including emergency department records, institutional databases, and self-reported surveys.

Four studies focused on general pickleball injuries, including emergency department data, national trends, and prevalence [4,5,7,18]. Four studies specifically addressed injuries to the lower extremity, foot, ankle, or Achilles tendon [6,8,10,15]. Two studies examined injury epidemiology [9,14], and two addressed treatment outcomes or return-to-play status [3,6]. Two studies focused on senior or elderly populations [1,2], and two employed systematic, scoping, or narrative review methodologies [11,16]. One study provided a comparative analysis by including tennis injuries alongside pickleball data [1].

Risk Factors 

Age:Age emerged as a consistent risk factor across nearly all studies [1-11,14-15]. The predominant population affected by pickleball injuries comprised individuals over the age of 50. Across studies reporting mean or median participant age, values clustered in the late-50s to mid-60s range, consistent with the sport's concentration among older recreational athletes. One study found that adults experienced a significantly higher frequency of lower extremity injuries compared to other age groups (p = 0.022) [14]. The concentration of injuries in this demographic reflects both the sport's popularity among older adults and potential age-related physiological vulnerabilities.

Gender: Gender-based findings were mixed across the eight studies that reported sex-disaggregated data. Six studies reported that males were more likely to sustain pickleball-related injuries, with injury rates of 50.4%, 53%, 58.6%, and 60.3% [3,6,7,9,15,18]. In contrast, two studies reported that females were more likely to be injured, with statistically significant results (p < 0.001 for both) [2,8]. One study reported that males were significantly more likely to sustain lower extremity injuries (p < 0.0001), while females were more likely to sustain upper extremity injuries (p < 0.0001) [14].

*Affected Anatomical Regions* 

The literature presented mixed findings regarding the most commonly affected anatomical regions. Among studies reporting injury location, four identified the lower extremity as the predominant site, and three found the upper extremity to be more commonly affected. Within the lower extremity, the ankle, calf, and lower back were the most frequently reported sites [3,6,7,9,16]. Lower extremity injuries accounted for 32.0% of injuries in one study [7] and 57.9% in another [9]. One imaging-based study reported 58% lower extremity involvement [16].

Upper extremity injuries were reported across a broader range of anatomical locations, including the radius (30%), wrist (12.7%), rotator cuff (11%), humerus (8%), and ulna (3%) [3,5,8,16]. This distribution may be influenced by specific injury types studied in each investigation, introducing reporting variability. Overall, the majority of reported injuries involved muscle and tendon tissues, accounting for 61.8% of cases in one cross-sectional study [2].

Injury Types

Sprains and strains and fractures were identified as the most commonly reported injury types across the included studies. Among nine studies reporting injury type prevalence, four identified sprains and strains as the most common injury [1,3,6,7], with reported rates ranging from 17% to 30.8% [2,3,5,7]. Males were significantly more likely to sustain sprains and strains compared to females (p < 0.0001) [1,14].

**Figure 2:**
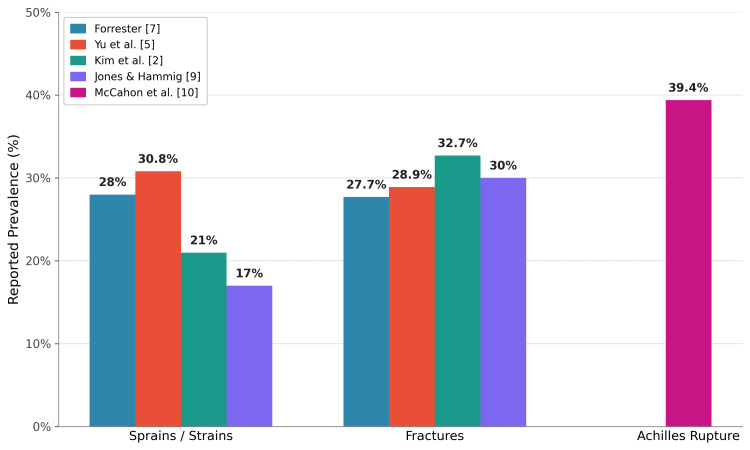
Injury type distribution across studies. Distribution of injury types across studies reporting usable percentages. Sprains and strains prevalence ranged from 17% to 30.8% [2,5,7,9], while fractures clustered more tightly between 27.7% and 32.7% [5,7-9]. Achilles tendon rupture was reported at 39.4% in one study focused on tendon injuries in elderly players [10]. Data are drawn from studies with heterogeneous populations and inclusion criteria; direct cross-study comparison should be interpreted with caution.

Four additional studies reported fractures as the most frequent injury type [4,5,9,14], with prevalence ranging from 27.7% to 32.7% [4,5,7,9]. Females were significantly more likely to sustain fractures (p < 0.0001; p = 0.021) [1,3,14]. One study found that Achilles tendon rupture was the most common injury, accounting for 39.4% of cases [10]. Other reported injuries included contusions [1], arthritis-related pain [3], and internal organ injuries (7.2%) [9], although these were less commonly observed (Figure 2).

Mechanisms of Injury 

Five studies examined mechanisms of injury. Falls emerged as the leading mechanism in three studies, with one reporting that falls were the primary cause of fractures (p < 0.001) [1,5,8]. One study combined falls, slips, trips, and dives into a broader instability category [1]. Two studies reported foot and ankle inversion as a common mechanism, with one highlighting this finding specifically among individuals aged 18 to 34 [5,15].

**Figure 3:**
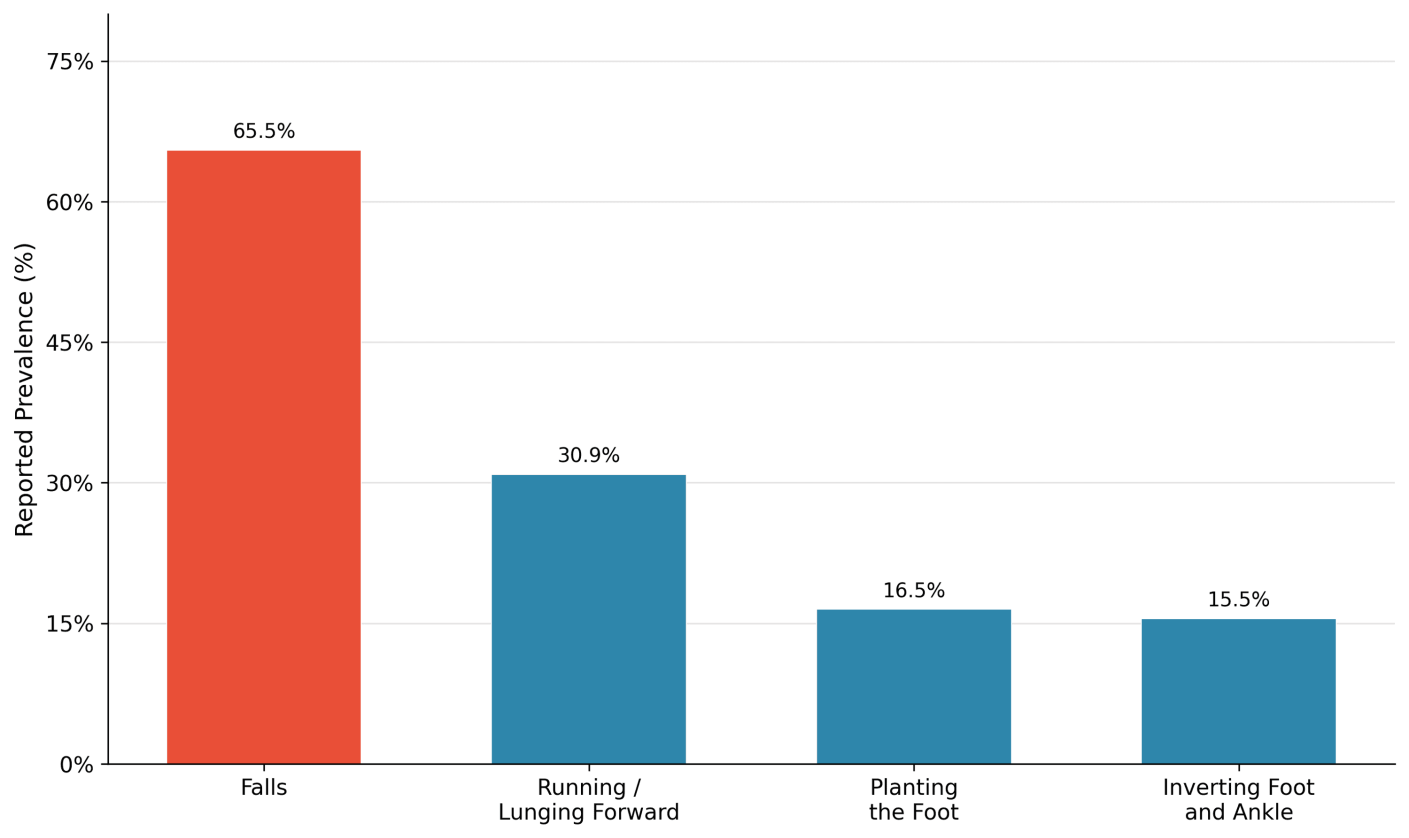
Mechanism of injury for lower extremity pickleball injuries. Reported mechanisms of injury. Falls were the dominant mechanism at 65.5% of injuries in one cross-sectional study of older players [2]. Running or lunging forward (30.9%), planting the foot (16.5%), and inverting the foot and ankle (15.5%) were reported in a study of foot and ankle injuries [15]. Data are drawn from different study populations; the falls data reflect a self-reported survey of older adults, while the biomechanical mechanisms reflect an institutional foot and ankle cohort.

Additional mechanisms reported across the literature included sudden changes in direction during play [6], running or lunging forward (30.9%) [15], planting the foot (16.5%) [15], and being struck by a paddle [5]. These findings collectively illustrate the biomechanical demands of pickleball, particularly the rapid acceleration, deceleration, and lateral movement patterns inherent to the sport [11] (Figure 3).

Discussion 

Overview 

This systematic review analyzed the growing body of literature on pickleball-related injuries, with a specific focus on lower-extremity involvement. The 15 included studies, spanning retrospective analyses, cross-sectional surveys, case series, and reviews, provide a multifaceted view of injury patterns in this rapidly expanding sport. The findings reveal recurring themes related to risk factors, injury patterns, and mechanisms that have important implications for clinical practice, injury prevention, and future research.

Age and Gender as Primary Risk Factors 

Age was the most consistent risk factor, with injuries concentrated among individuals aged 50 and older and a mean age of around 60 years [1-11,14-15]. This pattern is expected given the demographic profile of recreational pickleball participants, but also reflects age-related physiological vulnerabilities, including decreased tendon elasticity, reduced balance and proprioception, slower reaction times, and underlying degenerative changes that increase injury susceptibility [12,13]. As pickleball continues to grow in popularity among older adults [19], these findings highlight a pressing need for age-tailored injury prevention strategies, including structured warm-up protocols, proprioceptive training, footwear optimization, and targeted public health messaging [12,19].

Gender-based trends were variable but clinically important. The majority of included studies reported higher injury rates in males [3,6,7,9,15,18], while a smaller subset identified a significantly greater proportion of injuries in females [2,8]. One study noted that males were more prone to lower extremity injuries while females more commonly sustained upper extremity injuries [14]. These distinctions may reflect differences in playing style, biomechanics, bone mineral density, or fall mechanics [12,20]. Recreational programs should therefore consider sex-specific safety messaging. For older male players, emphasis should be placed on Achilles tendon loading awareness, eccentric calf strengthening, and graded warm-up progression. For older female players, emphasis should be placed on fall-prevention training and bone health screening, given the disproportionate fracture risk observed in the literature.

Injury Location and Tissue Involvement 

The literature presents a nuanced picture regarding anatomical distribution. Studies variably identified the lower extremity or the upper extremity as the predominant site of injury [2,3,5-9,16], likely reflecting differences in study design, data sources, and patient populations. Lower extremity injuries may be more closely associated with explosive court movements, such as pivoting, lunging, and directional changes, whereas upper extremity injuries may be associated with falls, collisions, or improper paddle mechanics [11].

Muscle and tendon tissues accounted for the majority of injuries in at least one dataset [2], highlighting the biomechanical demands of the sport and the importance of neuromuscular conditioning and load management. Overuse injuries, such as plantar fasciitis, Achilles tendinopathy, and stress-related conditions, are likely underrepresented in the current literature because patients with these conditions typically seek care in primary care or physical therapy settings rather than emergency departments. This reporting bias is compounded by the reliance of most included studies on NEISS and single-institution emergency records, and it should be considered when interpreting both injury type and incidence data.

Injury Type Trends 

Sprains and strains were the most commonly reported injury type, identified as predominant in four studies [1,3,6,7], with reported rates ranging from 17% to 30.8% [2,5,7,9]. Males were significantly more likely than females to sustain sprains and strains (p < 0.0001) [1,14]. Fractures were the predominant injury type in a separate group of studies, with prevalence ranging from 27.7% to 32.7% [2,5,7,9]. Females were significantly more likely than males to sustain fractures (p < 0.0001; p = 0.021) [1,3,14], suggesting that anatomical and physiological differences, including lower bone mineral density, may contribute to this pattern.

Achilles tendon rupture was identified as the most prevalent injury in one study focused on tendon injuries in elderly players [10]. This underscores the unique biomechanical demands placed on the lower limb during pickleball, particularly among aging adults who may already have underlying tendon degeneration. Together, these findings suggest that injury type is influenced not only by the demands of the sport but also by player-specific factors, including age, gender, and musculoskeletal resilience.

Mechanisms of Injury and Sport-Specific Factors

Falls were the leading cause of injury in three of the five studies that examined mechanism, particularly in relation to fractures [1,5,8]. Additional mechanisms included sudden directional changes [6], lunging or running forward [15], foot planting [15], and foot and ankle inversion [5,15]. One study identified paddle contact as a less common but relevant mechanism [5].

These findings emphasize the physical demands of pickleball, especially in older or recreational players who may not routinely train for lateral agility or proprioceptive control. An important and understudied area is the role of sport-specific court mechanics in injury risk. Unlike other racquet sports, pickleball is played on a smaller court requiring quick reflexes and abrupt start-stop movements within a confined space. The “kitchen” or no-volley zone, a seven-foot area near the net where volleying is prohibited, often forces players into reaching, bending, lunging, and sudden halting movements that increase injury exposure. Players should be coached on proper lunging mechanics and weight distribution to maintain stability in this zone, and instructional sessions for recreational players should emphasize controlled footwork during transitions in and out of the no-volley area.

Court surface characteristics likely also contribute to injury risk. Surface friction varies considerably between outdoor settings (typically acrylic-coated asphalt or concrete) and indoor settings (gymnasium wood or sport-tile), with implications for both slip-induced falls and sudden-stop inversion injuries. While direct comparative data on the coefficient of friction across pickleball-specific surfaces remain limited, routine inspection of court surfaces for traction consistency is a reasonable operational recommendation for facility operators aiming to reduce slip-and-fall events, which dominate the injury data.

Comparison With Other Racquet Sports

Comparison with other racquet sports, though limited, suggests that pickleball carries a distinct risk profile. Weiss et al. [1] noted that senior pickleball injuries more frequently involved slips, trips, and falls than tennis injuries, consistent with the sport's compressed court and reactive play. Changstrom et al. [18] similarly reported differing patterns across racquet and paddle sports, with radius fracture prominent in combined datasets largely driven by upper-extremity events. These comparisons support the view that pickleball-specific biomechanics, rather than racquet sport exposure alone, shape the injury patterns observed in the present review.

Practical Recommendations

The findings of this review support a shift from awareness to action. Clinicians, community organizations, and facility operators should prioritize the implementation of structured injury prevention programs. Specifically, programs should incorporate dynamic warm-up protocols, proprioceptive and balance training, sex-specific safety messaging, and sport-specific footwear guidance. Routine court surface inspection and player education on no-volley zone mechanics are additional practical interventions that may reduce the falls and inversion injuries that dominate the literature. Recreational facilities should consider implementing brief mandatory safety orientations for new players, with content tailored by age and sex. Table 2 summarizes the principal modifiable risk factors identified in this review paired with targeted practical recommendations.

**Table 2:** Modifiable risk factors. Modifiable risk factors identified in this systematic review and corresponding practical recommendations for players, clinicians, and facility operators.

Modifiable risk factor	Practical recommendation
Falls and postural instability	Proprioceptive and balance training (e.g., tai chi, single-leg stance drills) for 10 to 15 minutes, two to three times weekly for players aged 50 and older.
Foot and ankle inversion	Neuromuscular ankle conditioning and court-specific footwear providing adequate lateral support.
Achilles tendon overload (older male players)	Graded eccentric calf loading program; dynamic calf warm-up prior to play.
Fracture risk (older female players)	Bone health screening when clinically indicated; fall-prevention training focused on controlled landing and recovery techniques.
Insufficient pre-activity warm-up	Mandatory 5 to 10 minute dynamic warm-up at community and club facilities.
No-volley zone biomechanics	Coached lunging and deceleration drills; instruction on controlled transitions into and out of the no-volley area.
Court surface variability (indoor vs outdoor)	Regular surface inspection by facility operators; clear signage when surface friction or conditions change.
Inadequate player education	Brief mandatory safety orientation for new recreational players, with content tailored by age and sex.

Limitations 

Several limitations should be considered. First, the review protocol was not prospectively registered in PROSPERO, which limits transparency regarding deviations from the original search plan. Second, no formal risk-of-bias assessment tool, such as the Newcastle-Ottawa Scale (NOS) [21], was applied to individual studies. Although each study was qualitatively evaluated for methodological transparency, clarity of outcome definitions, and relevance during inclusion review, the absence of structured scoring limits the ability to weight findings by methodological rigor. We agree with this limitation and recognize that future reviews in this rapidly evolving literature should incorporate a formal NOS or an equivalent risk-of-bias assessment. Third, certainty of evidence was not graded using the Grading of Recommendations, Assessment, Development, and Evaluations (GRADE) framework. GRADE is primarily designed for meta-analyses and bodies of evidence addressing defined clinical questions; the descriptive, heterogeneous nature of the current pickleball injury literature, dominated by retrospective observational and database-derived studies, does not yet support meaningful outcome-level certainty grading. Application of GRADE will become more feasible as prospective and homogeneous studies accumulate.

Only a subset of included studies specifically addressed lower extremity injuries; the majority reported on pickleball injuries broadly, requiring the extraction of relevant data from within broader analyses. This introduces potential reporting bias. Most studies relied on emergency department data from the NEISS database in the United States, which likely overrepresents severe injuries requiring emergency care and underrepresents less serious injuries managed in outpatient or community settings. Notably, overuse conditions, such as plantar fasciitis and tendinopathies, are likely underreported because these patients tend to present to primary care or physical therapy rather than emergency departments, a limitation compounded by the geographic skew toward United States data. Several studies were limited to single institutions, restricting generalizability. One study reported losing more than half of its population to follow-up [6]. Some studies included data from related racquet sports, which may obscure pickleball-specific findings [1,6,18]. Self-reported data in one study [2] and exclusive focus on foot and ankle injuries in another [15] further limit comparability.

The inclusion of one narrative review [11] represents a deviation from conventional systematic review methodology. This study was retained because it provided extractable mechanistic data on sport-specific injury patterns not reported in the primary research studies; this decision is transparently reported to allow readers to weigh its implications.

Future Directions 

As pickleball continues to grow, prospective cohort studies collecting data beyond emergency departments, for example, at competitions and community recreation centers, would provide a more representative picture of injury prevalence and severity. Biomechanical studies examining the specific demands of pickleball court mechanics, including the role of court size, the no-volley zone, paddle weight, and surface friction, are needed to understand how these factors contribute to injury risk. Development and evaluation of structured injury prevention programs, including warm-up protocols, balance training, and footwear recommendations, represent important translational opportunities.

## Conclusions

This systematic review of 15 studies demonstrates that lower extremity injuries in adult pickleball players are common, predominantly involve sprains and strains and fractures, and disproportionately affect adults over the age of 50. Falls and rapid directional movements are the primary injury mechanisms. Gender-based trends indicate that males are more susceptible to soft tissue injuries while females are more prone to fractures. The unique court mechanics of pickleball, including confined playing space and the no-volley zone, create biomechanical demands that contribute to injury risk. Clinicians, community organizations, and facility operators should prioritize the implementation of structured injury prevention programs. Specifically, these programs should incorporate dynamic warm-up protocols, proprioceptive and balance training, sex-specific safety messaging, sport-specific footwear guidance, routine court surface inspection, and coached instruction on no-volley zone mechanics. As the sport continues to expand, prospective epidemiological studies and biomechanical investigations are needed to strengthen the evidence base for prevention and clinical management.
